# Avian cell line - Technology for large scale vaccine production

**DOI:** 10.1186/1753-6561-5-S8-P52

**Published:** 2011-11-22

**Authors:** Barbara Kraus, Simone von Fircks, Simone Feigl, Sabrina M Koch, Daniel Fleischanderl, Katherine Terler, Mitra Dersch-Pourmojib, Christian Konetschny, Leopold Grillberger, Manfred Reiter

**Affiliations:** 1 Baxter Innovations GmbH, Uferstrasse 15, 2304 Orth/Donau, Austria

## Introduction

The establishment of an immortalized continuous cell line derived from quail cells was undertaken by Baxter in order to develop a new cell line platform for vaccine production that is free of genetically modifying sequences.

Important vaccines and viral vectors are still produced in embryonated chicken eggs or primary chicken embryo fibroblasts. However, the substitution of primary cells by a continuous cell line has several advantages. Although primary avian tissue for virus replication is provided by specific pathogen-free (SPF) production plants, sterility during vaccine production on embryonated eggs is difficult to guarantee, and the constant risk of contamination necessitates the addition of antibiotics. In addition, the supply of embryonated SPF eggs could be a limiting factor in vaccine production if increased amounts are demanded by the vaccine manufacturers, e. g. in case of a pandemic outbreak.

The same is true for approaches where primary fibroblast monolayer cultures are used. Thus, avian cell lines have become a modern option for vaccine manufacturing and will definitely replace egg and primary fibroblast technology.

## Cell Line Development and Characterization

Here we describe the development of a continuous avian cell line from quail embryos into serum-free suspension culture and its manufacturing potential for several different vaccines.

**Figure 1:**
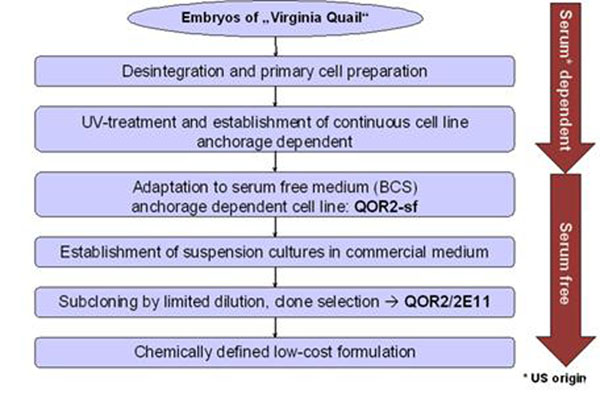
Schematic overview of the cell line history

Briefly, embryos of *Colinus virginianus virginianus* were disintegrated and trypsinized to obtain a primary culture that grew adherently in serum-containing medium. After UV treatment with a specific dose [1], immortalized cells were expanded and subsequently adapted to serum-free conditions (QOR2-sf) and growth in suspension using a chemically defined medium. Subcloning resulted in the isolation of clone QOR2/2E11, which was selected as the lead clone for development of vaccine production processes. Then, quality-controlled cell banks were established (Figure 1). The cell clone QOR2/2E11 is grown in a chemically defined, animal component-free medium (Baxter proprietary formulation).

Quality control (QC) testing was performed at different stages during cell line development. Extended characterization according to relevant guidelines (Table 1) showed that the cells are free of adventitious agents and F-Pert negative. Tumorigenicity testing performed in BALB/c nude mice indicated no tumorigenic effect. Accordingly, the cells fulfill all the critical regulatory requirements.

Thus, the developed subclone QOR2/2E11 was considered to be qualified as source material for Good Manufacturing Practice (GMP) cell bank production. A pre master cell bank and cell bank derivates were produced in compliance with the current GMP regulations and are currently available.

**Table 1:** List of guidelines according to which the avian cell line was tested

Organization	Number	Title
**European Directorate for the quality of Medicines & HealthCare**	European Pharmacopoeia. (EP) 2.6.16.	Tests for extraneous agents in viral vaccines for human use
	
	EP 5.2.3.	Cell substrates for the production of vaccines for human use

**The International Conference on Harmonisation of Technical Requirements for Registration of Pharmaceuticals for Human Use (ICH)**	Q5A (R1)-1999	Viral safety evaluation of biotechnology products derived from cell lines of human of animal origin
	
	Q5D-1997	Derivation and characterization of cell substrates used for production of biotechnological/biological products
	
	Q7A-2000	Good manufacturing practice guide for active pharmaceutical ingredients

**Center for Biologics Evaluation & Research (CBER)**	----	Points to Consider in the Characterization of Cell Lines Used to Produce Biologicals (1993)
	
	----	Guidance for Industry, Characterization and Qualification of Cell Substrates and Other Biological Materials Used in the Production of Viral Vaccines for Infectious Disease Indication (February 2010)

**Code of Federal Regulations (CFR)**	9CFR113.47	Detection of extraneous agents viruses by fluorescent antibody technique
	
	21CFR610.18	General biological products standards

## Scalable live virus production in QOR avian cells

For process development purposes, modified vaccinia Ankara (MVA) virus was used as a model virus. MVA virus is a highly attenuated strain of vaccinia virus belonging to the Poxviridae family that was produced by over 500 passages in chicken embryo fibroblasts. MVA has lost about 10 % of the vaccinia dsDNA genome and consequently cannot replicate in primate and human cells. Its complex genome of circa 200 kb allows the insertion of large exogenous DNA inserts. Therefore, MVA serves as a versatile live vector for the development of human vaccines against diverse disease targets, such as malaria and cancer, for which conventional approaches have so far failed [2].

**Figure 2:**
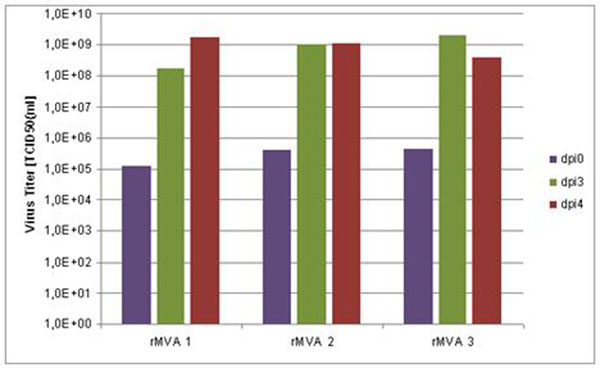
Growth Kinetics of rMVA viruses on QOR2/2E11

Using the QOR cell line, high product titers can be achieved with a broad range of viruses such as wild-type MVA, recombinant MVA (rMVA) strains, influenza and flaviviruses. Figure 2 shows the growth of virus in 200 ml spinner flask cultures using different rMVA constructs. The experiments were performed with a starting cell density of about 2 x 10^6^ cells per ml and a multiplicity of infection between 1.0 and 0.1. Over the course of four days post infection (dpi), virus titers of ≥ 1 x 10^9^ TCID_50_ per ml were obtained with every rMVA construct tested. Constant cell performance up to 10L bioreactors (laboratory scale), where cell densities ≥ 2 x 10^6^ cells per ml were achieved, has been confirmed and scalability to 100 - 1000L bioreactors is currently under evaluation.

Furthermore, this avian cell line can serve as a control cell line with different model viruses in QC tests for adventitious agents.

## Conclusion

A scalable technology for the production of live and attenuated vaccines based on the qualified avian cell line QOR2/2E11 has been established. The markedly advantages are:

• Stable, continuous avian cell line established without the introduction of foreign genes

• Post production cells are not tumorigenic in animal model

• Suspension growth in low-cost chemically defined medium

• TCID_50_ titers ≥ 1 x 10^9^ for several rMVA constructs tested
